# Lymph node metastases in middle and upper mediastinum of Siewert type II adenocarcinoma: A real‐world retrospective study

**DOI:** 10.1002/cam4.6919

**Published:** 2024-03-11

**Authors:** Peng Luo, Xiankai Chen, Yafan Yang, Ruixiang Zhang, Xiaozheng Kang, Jianjun Qin, Xiuzhu Qi, Yin Li

**Affiliations:** ^1^ Department of Thoracic Surgery, National Cancer Center, National Clinical Research Center for Cancer, Cancer Hospital Chinese Academy of Medical Sciences and Peking Union Medical College Beijing China; ^2^ Department of Ultrasound Fudan University Shanghai Cancer Center Shanghai China; ^3^ Department of Oncology, Shanghai Medical College Fudan University Shanghai China

**Keywords:** esophagogastric junction, lymphadenectomy, mediastinal lymph node, metastases, siewert type II

## Abstract

**Objective:**

To explore whether the upper and/or middle mediastinal nodes (UMMN) should be dissected in Siewert type II adenocarcinoma (AC) according to the incidence of lymph node metastasis. Additionally, to investigate the association between the length of esophageal involvement (LEI) and the UMMN metastases.

**Methods:**

A cohort with Siewert type II AC who were operated on by a surgical team that routinely treated esophagogastric junction (EGJ) tumors with esophagectomy and extended lymphadenectomy were assessed retrospectively. The primary endpoint of the research was the metastasis rate of UMMN.

**Results:**

A total of 94 patients with EGJ tumor from July 2018 to September 2022 were enrolled. Station 106recR (6.4%, 6/94) was the only station among upper mediastinal nodes (UMN) that presented positive nodes. Middle mediastinal nodes (MMN) metastases of station 107, 109 and station 108 were 2.1% (2/94) and 5.0% (4/80), respectively. Among the 11 patients with MMN or UMN metastases, 63.6% (7/11) had lesser than seven metastatic nodes, and 54.5% (6/11) had a pathological N stage ≤2. LEI >3 cm (*p* = 0.042) showed a higher risk for MMN metastases in univariable logistic analysis. However, no independent risk factor for mediastinal node metastases was detected.

**Conclusion:**

This study demonstrated that the incidence of positive MMN and UMN is relatively low in resectable Siewert type II AC, which indicated that it is not necessary to perform a routine dissection upon these stations. LEI >3 cm might be associated with higher risk for mediastinal node metastasis. Certain patients could benefit from extended lymphadenectomy since most of the patients with positive MMN or UMN have a limited number of metastatic nodes.

## 1 INTRODUCTION

Adenocarcinoma (AC) of esophagogastric junction (EGJ) has become a growing health concern in the past few decades^1^, ^2^, ^3^ due to its rising incidence. The Siewert classification of EGJ AC raised by Siewert and Holsher in 1987 is a widely accepted and practical classification system.^4^, ^5^ Existing evidence revealed that Siewert type I (located 1 to 5 cm above the EGJ) and type III (located 2 to 5 cm below the EGJ) tumor were distinct entities requiring different surgical options.^6^, ^7^, ^8^, ^9^ However, as for Siewert type II (located 1 cm above to 2 cm below the EGJ) tumor, which is known as the true AC of the cardia, a consensus upon the appropriate surgical approaches and the optimal extent of lymph node (LN) dissection have not been reached.^3^, ^10^ In effect, for the same Siewert type II patient, gastric surgeons prefer the transhiatal approach (TH), while thoracic surgeons favor the transthoracic approach (TT). Different approaches determine different extent of lymph node dissection. In particular, TH increases the risk of leaving positive nodes in the mediastinum.^11^

So far, studies investigating the middle mediastinal nodes (MMN) and upper mediastinal nodes (UMN) dissection in Siewert type II AC have been rarely reported.^12^, ^13^, ^14^, ^15^ Therefore, the metastases of MMN and UMN for Siewert type II AC in real‐world situations require further exploration.

At the Department of Thoracic Surgery of National Cancer Center of China, minimally invasive Mckeown esophagectomy has been routinely applied by one of the Authors (YL) and his team to treat patients with resectable Siewert type II tumor since 2018. The operation encompassed extended lymphadenectomy, and mediastinal nodes (including MMN and UMN) dissection were routinely performed.

The present study endeavors to address the query regarding whether the upper and/or middle mediastinal nodes (UMMN) should be dissected based on the incidence of lymph node metastasis, and to investigate if the length of esophageal involvement (LEI) predicted higher risk of UMMN metastases using a cohort of Siewert type II cancer from the National Cancer Center of China.

## 2 METHODS

One hundred fifty‐nine patients with resectable EGJ carcinoma who underwent esophagectomy by one surgical team at the thoracic department of the National Cancer Center of China from July 2018 to September 2022 were retrospectively collected from the institutional database. The study was approved by the Institutional Review Board of National Cancer Center, and informed consent was waived.

The exclusion criteria were as follows^1^: pathological diagnosis of non‐adenocarcinoma,^2^ tumor of Siewert type I or type III,^3^ history of previous or concomitant malignancy,^4^ surgery not performed by the surgical team (even if MMN or UMN were dissected),^5^ surgery type other than minimally invasive Mckeown esophagectomy, and^6^ incompleteness of clinicopathological data. Patients with pathological identification of other concurrent differentiation, such as adenosquamous carcinoma and neuroendocrine carcinoma, were excluded from this study. Preoperative chemotherapy was administered to patients with clinical T stage ≥3 or with clinical positive nodes. The primary endpoint of the research was the metastasis rate of UMMN.

The distance from proximal margin or epicenter of tumor to the EGJ was measured by endoscopy or gross pathology. The extended lymphadenectomy included mediastinal nodes and abdominal nodes. The upper mediastinal nodes consisted of station 105 (upper thoracic para‐esophageal node), station 106recR (right recurrent laryngeal nerve node), station 106recL (left recurrent laryngeal nerve node), and station 106tbL (left tracheobronchial node). Middle mediastinal nodes were station 107 (subcarinal node), station 108 (middle thoracic para‐esophageal node), station 109 (left and right main bronchus node). Lower mediastinal nodes contained station 110 (lower thoracic para‐esophageal node) and station 111 (supra‐diaphragmatic node). Abdominal nodes were station 1 (right paracardial node), station 2 (left paracardial node), station 3 (lesser curvature node), station 7 (node along the trunk of left gastric artery), station 8a (anterosuperior node along the common hepatic artery) and station 11 (splenic artery node). Station 107 and station 109 were resected and counted as a whole owing to their close anatomical relationship. Likewise, station 7, station 8a, and station 11 followed the same procedure. All continuous variables were expressed as median and range. Risk factors associated with lymph node metastases (LNM) were explored using logistic regression analysis. Variables with *p*‐values <0.20 on univariate analysis were included in the multivariable analysis. *p* < 0.05 were considered statistically significant.

## 3 RESULTS

**FIGURE 1:**
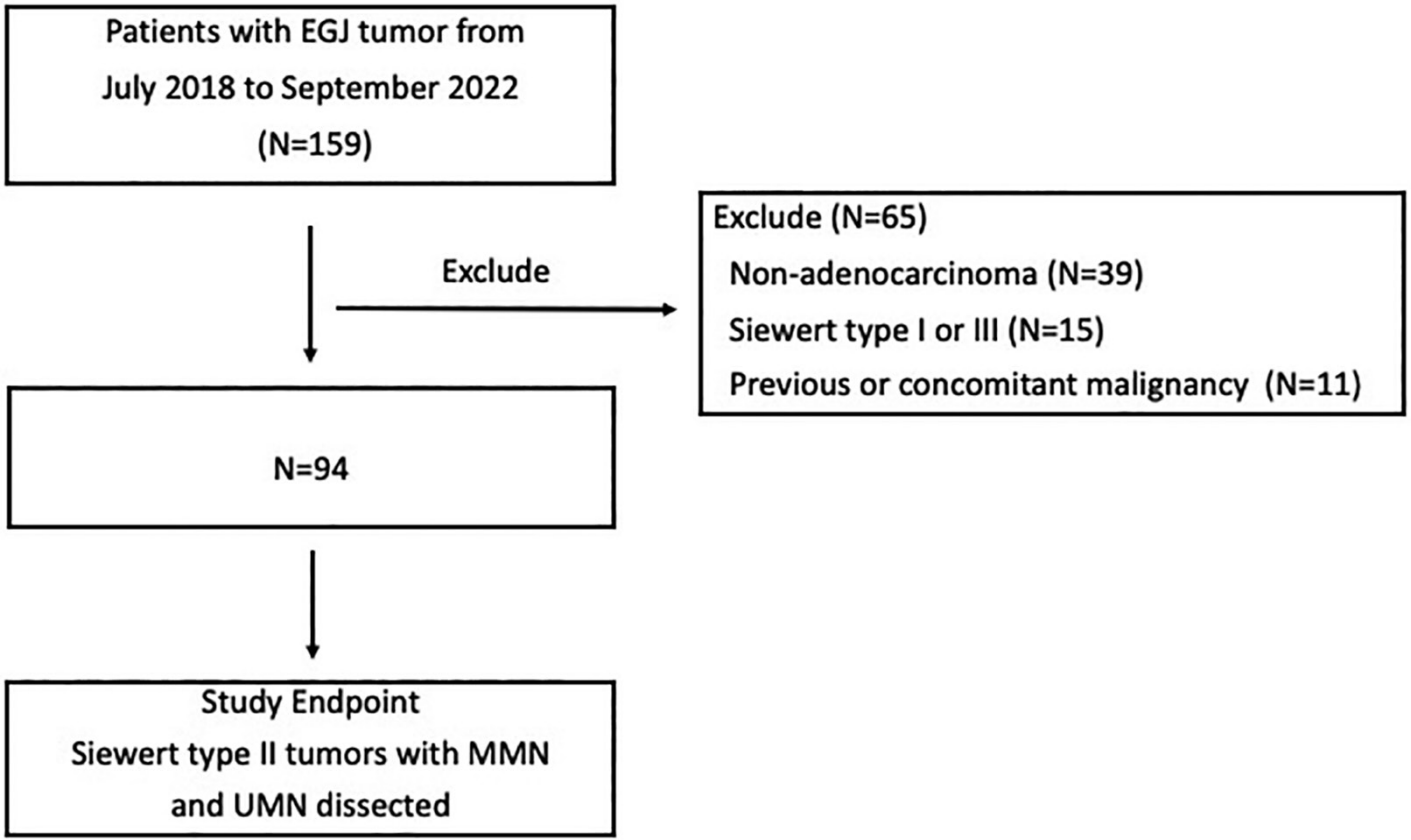
Flowchart of patients.

From June 2018 to September 2022, a total of 159 patients diagnosed with EGJ carcinoma received surgery by the same surgical group at the Department of Thoracic Surgery of National Cancer Center of China. Of these, 65 patients were excluded from the study. Finally, a total of 94 patients with Siewert II AC were enrolled (Figure 1).

Demographic and clinicopathological characteristics of patients are summarized in Table 1. The median age was 62 years (range, 33–82 years). The majority of patients were men (86.2%), diagnosed with clinical T3 disease (53.2%), had poorly differentiated tumor (59.6%), and underwent conventional minimally invasive esophagectomy (cMIE) (88.3%), with Lauren classification of intestinal type (57.4%). Fifty‐two patients (55.3%) received preoperative chemotherapy (55.3%). The majority of patients received oxaliplatin plus capecitabine (40 patients) and oxaliplatin plus S‐1 (eight patients).

**TABLE 1:** Characteristics of 94 patients.

Gender	
Female	13 (13.8%)
Male	81 (86.2%)
Age (y)	
Median (range)	62 (33–82)
BMI	
Median (range)	24.6 (15.5–34.4)
Tumor length (cm)	
Median (range)	6.0 (1.0–11.0)
LEI (cm)	
Median (range)	2.0 (0–6.0)
Clinical T stage	
T1a	3 (3.2%)
T1b	16 (17.0%)
T2	13 (13.8%)
T3	50 (53.2%)
T4a	12 (12.8%)
Clinical N stage	
N−	39 (41.5%)
N+	55 (58.5%)
Tumor grade	
Well‐differentiated	4 (4.3%)
Moderately differentiated	34 (36.2%)
Poorly differentiated	56 (59.6%)
Lauren classification	
Intestinal type	54 (57.4%)
Diffuse type	12 (12.8%)
Mixed type	24 (25.5%)
Unknown	4 (4.3%)
Preoperative chemotherapy	
Yes	52 (55.3%)
No	42 (44.7%)
Surgical type	
RAMIE	11 (11.7%)
cMIE	83 (88.3%)
Operation duration (min)	
Median (range)	230 (130–483)
Pathologic T stage	
0	3 (3.2%)
tis	1 (1.1%)
1a	8 (8.5%)
1b	16 (17.0%)
2	16 (17.0%)
3	39 (41.5%)
4a	11 (11.7%)
Pathologic N stage	
0	43 (45.7%)
1	33 (35.1%)
2	12 (12.8%)
3	17 (18.1%)
Number of retrieved nodes	
Median (Range)	35 (13–71)
Number of retrieved UMN	
Median (Range)	6 (1–30)
Number of retrieved MMN	
Median (Range)	6 (1–18)
Nerve invasion	
No/unknown	50 (53.2%)
Yes	44 (46.8%)
Vessel invasion	
No/unknown	57 (60.6%)
Yes	37 (39.4%)

Abbreviations: BMI, body mass index; cMIE, conventional minimally invasive esophagectomy; LEI, length of esophageal involvement; MMN, middle mediastinal nodes; N−, without clinical positive nodes; N+, with clinical positive nodes; RAMIE, robot‐assisted minimally invasive esophagectomy; UMN, upper mediastinal nodes.

The median tumor size was 6.0 cm (range, 1.0–11.0 cm). The median length of esophageal involvement was 2.0 cm (range, 0–6.0 cm). The median number of retrieved nodes was 35 (range, 13–71). The median number of retrieved UMN and MMN was 6 (range, 1–30) and 6 (range, 1–18), respectively.

**FIGURE 2:**
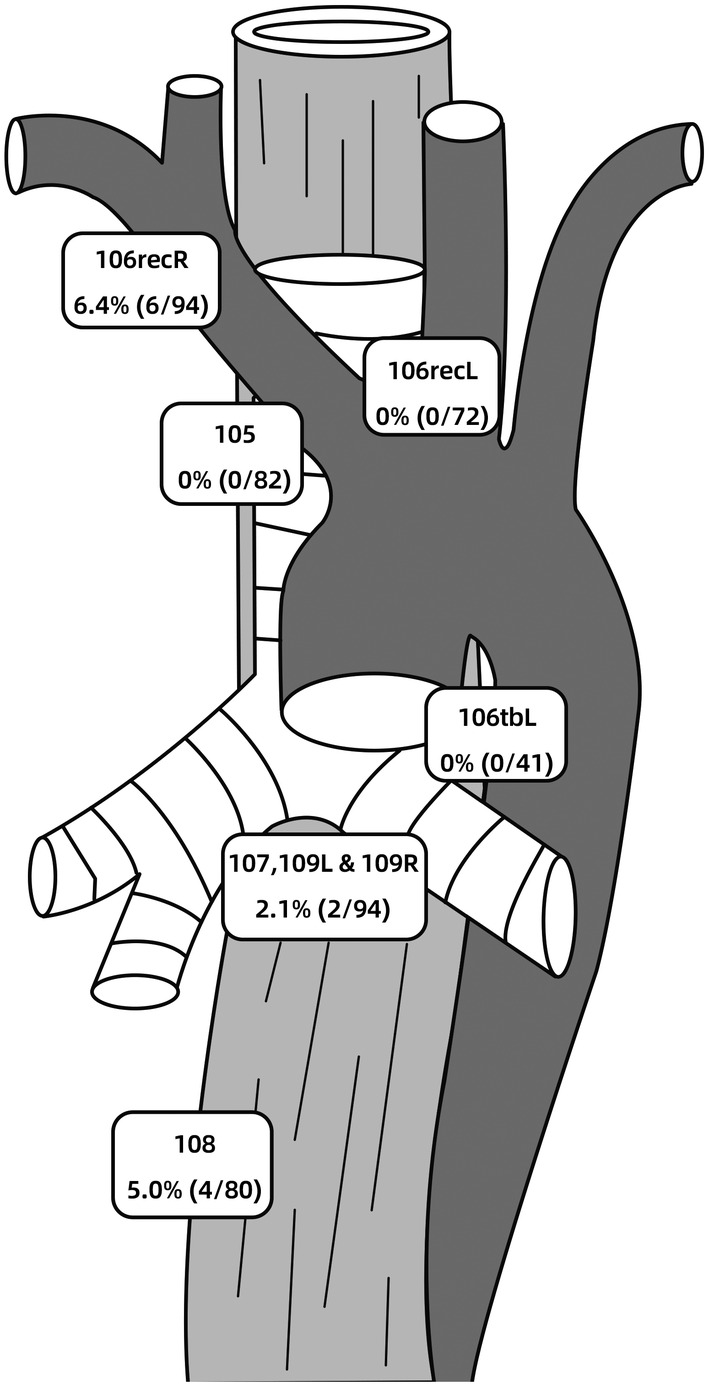
Metastasis rates in the upper and middle mediastinal nodes of the 94 patients with Siewert type II adenocarcinoma.

Generally, 54.3% (51/94) of patients had lymph node metastases in the resected specimens. The metastasis rates of each station are summarized in Table 2. The overall metastasis rate of abdominal nodes was 51.1% (48/94), and station 7, 8a, and 11 were as high as 38.3% (36/94). The overall metastasis rates of upper, middle, and lower mediastinal nodes were 6.4% (6/94), 6.4% (6/94), and 12.9% (11/85), respectively. Among mediastinal nodes, station 110 showed the highest metastasis rate of 9.5% (8/84). Only 2 (2.1%, 2/94) cases presented with station 107, 109 metastases. Among the four stations of UMN, station 106recR (6.4%, 6/94) was the only station with positive nodes (Figure 2). One patient had both positive UMN and positive MMN. 11.7% (11/94) of patients had UMMN metastases. Clinical T stage ≥3, poor differentiation, tumor length >4 cm, and LEI >3 cm seemed to have higher metastasis rate for UMMN.

**TABLE 2:** Lymph node metastasis rates of the 94 patients.

Nodal station	Overall rate	Clinical T stage		Tumor grade		Preoperative chemotherapy		Tumor length		Length of esophageal involvement	
		≥3	<2	Poor	Well/Moderate	Yes	No	≤4 cm	>4 cm	≤3 cm	>3 cm
UMN	6.4% (6/94)										
105	0% (0/82)										
106tbl	0% (0/41)										
106recL	0% (0/72)										
106recR	6.4% (6/94)	9.7% (6/62)	0% (0/32)	8.9% (5/56)	2.6% (1/38)	9.6% (5/52)	2.4% (1/42)	3.1% (1/32)	8.1% (5/62)	5.1% (3/59)	8.6% (3/35)
MMN	6.4% (6/94)										
107, 109	2.1% (2/94)	3.2% (2/62)	0% (0/32)	3.6% (2/56)	0% (0/38)	3.8% (2/52)	0% (0/42)	0% (0/32)	3.2% (2/62)	0% (0/59)	5.7% (2/35)
108	5.0% (4/80)	5.6% (3/54)	3.8% (1/26)	8.9% (4/45)	0% (0/35)	6.1% (3/49)	3.2% (1/31)	2.8% (1/36)	5.6% (3/54)	2.0% (1/49)	9.7% (3/31)
LMN	12.9% (11/85)										
110	9.5% (8/84)	14.3% (8/56)	0% (0/28)	16.7% (8/48)	0% (0/36)	12% (6/50)	5.9% (2/34)	0% (0/28)	14.3% (8/56)	0% (0/53)	25.8% (8/31)
111	3.5% (3/85)	3.6% (2/56)	3.4% (1/29)	4.2% (2/48)	2.7% (1/37)	0% (0/49)	8.3% (3/36)	0% (0/30)	5.5% (3/55)	1.9% (1/53)	6.3% (2/32)
AN	51.1% (48/94)										
1	17.9% (15/84)	21.4% (12/56)	10.7% (3/28)	27.1% (13/48)	5.6% (2/36)	22.0% (11/50)	11.8% (4/34)	10.7% (3/28)	21.4% (12/56)	13.2% (7/53)	25.8% (8/31)
2	14.7% (11/75)	17.6% (9/51)	8.3% (2/24)	22.7% (10/44)	3.2% (1/31)	19.1% (9/47)	7.1% (2/28)	11.5% (3/26)	16.3% (8/49)	13% (6/46)	17.2% (5/29)
3	23.4% (22/94)	27.4% (17/62)	15.6% (5/32)	32.1% (18/56)	10.5% (4/38)	30.8% (16/52)	14.3% (6/42)	15.6% (5/32)	27.4% (17/62)	20.3% (12/59)	28.6% (10/35)
7, 8a, 11	38.3% (36/94)	50.0% (31/62)	15.6% (5/32)	57.1% (32/56)	10.5% (4/38)	48.1% (25/52)	26.2% (11/42)	25.0% (8/32)	45.2% (28/62)	37.3% (22/59)	40.0% (14/35)

Abbreviations: 1, right paracardial node; 105, upper thoracic para‐esophageal node; 106recL, left recurrent laryngeal nerve node; 106recR, right recurrent laryngeal nerve node; 106tbL, left tracheobronchial node; 107, subcarinal node; 108, middle thoracic para‐esophageal node; 109, left and right main bronchus node; 11, splenic artery node; 110, lower thoracic para‐esophageal node; 111, supra‐diaphragmatic node; 2, left paracardial node; 3, lesser curvature node; 7, node along the trunk of left gastric artery; 8a, anterosuperior node along the common hepatic artery; AN, abdominal node; LMN, lower mediastinal node; MMN, middle mediastinal node; Moderate, moderately differentiated; Poor, poorly differentiated; UMN, upper mediastinal node; Well, well‐differentiated.

In the univariable logistic regression analyses, LEI >3 cm (*p* = 0.042) was a significant risk factor for MMN metastases. LEI >3 cm (*p* = 0.065) and poor differentiation (*p* = 0.052) showed higher risk for UMMN metastases. However, the discrepancy did not reach a significant level. No risk factors were detected for UMN metastases. According to multivariable logistic regression analyses, there were no independent risk factors for mediastinal node metastases (Table 3).

**TABLE 3:** Univariate and multivariate logistic analyses of lymph node metastasis in mediastinum.

Variables	UMN (6/94)				MMN (6/94)				UMMN (11/94)			
	Univariate	Multivariate			Univariate	Multivariate			Univariate	Multivariate		
	*p*‐Value	OR	95% CI	*p*	*p*‐Value	OR	95% CI	*p*	*p*‐Value	OR	95% CI	*p*
Clinical T stage												
<3 vs. ≥3	0.998				0.371				0.096	0.51	0.05–5.34	0.583
Tumor grade												
Well/Moderate vs. Poor	0.248				0.998				0.052	0.16	0.02–1.39	0.097
Preoperative chemo												
No vs. Yes	0.187				0.187	0.36	0.04–3.43	0.375	0.078	0.44	0.08–2.53	0.357
Tumor length												
≤4 cm vs. >4 cm	0.371				0.371				0.251			
LEI												
≤3 cm vs. >3 cm	0.508				0.042	0.13	0.01–1.18	0.070	0.065	0.47	0.10–1.72	0.225

Abbreviations: CI, confidence interval; LEI, length of esophageal involvement; MMN, middle mediastinal nodes; Moderate, moderately differentiated; OR, odds ratio; Poor, poorly differentiated; Preoperative Chemo, preoperative chemotherapy; UMMN, upper and/or middle mediastinal nodes; UMN, upper mediastinal nodes; Well, well‐differentiated.

The detailed information of 11 patients with MMN or UMN metastases is listed in Table 4. Notably, 63.6% (7/11) had the number of positive nodes ≤7. Moreover, 54.5% (6/11) had pathological N stage ≤2. Especially, 106recR was found to be the only positive station in one patient.

**TABLE 4:** Clinicopathological characteristics of 11 cases with MMN or UMN metastases.

Patient	Gender	Age	Tumor Length	LEI	cT	Preoperative chemotherapy	Mandard Score	Tumor Grade	Pathologic T stage	Pathologic N stage	Station 108 (positive/retrieved)	Station 107, 109 (positive/retrieved)	Station 106recR (positive/retrieved)	Overall positive nodes	Overall retrieved nodes
1	Male	65	7 cm	4 cm	4a	No	/	Po	3	3	/	0/9	1/4	16	31
2	Female	65	5 cm	2 cm	3	Yes	4	Po	3	3	0/1	0/4	1/6	7	35
3	Male	58	3 cm	2 cm	3	Yes	2	Mo	1b	1	0/2	0/3	2/4	2	35
4	Male	55	8 cm	4.5 cm	4a	Yes	4	Po	3	1	0/1	0/1	1/4	2	38
5	Male	76	4.5 cm	0.8 cm	3	Yes	4	Po	2	2	0/1	0/9	1/8	4	49
6	Male	62	7 cm	4 cm	3	Yes	4	Po	3	3	0/2	3/3	2/12	10	66
7	Male	61	7 cm	4 cm	3	Yes	4	Po	4a	3	0/1	1/6	0/4	25	61
8	Male	49	5 cm	3 cm	4a	Yes	3	Po	3	2	1/1	0/5	0/4	4	43
9	Male	59	6 cm	4 cm	3	Yes	4	Po	3	2	1/1	0/2	0/1	4	33
10	Male	69	8 cm	4 cm	3	No	/	Po	4a	2	1/4	0/4	0/4	4	34
11	Male	71	3 cm	1 cm	2	Yes	4	Po	4a	3	3/3	0/1	0/1	11	39

Abbreviations: 106recR, right recurrent laryngeal nerve node; 107, subcarinal node; 108, middle thoracic para‐esophageal node; 109, left and right main bronchus node; cT, clinical T stage; LEI, length of esophageal involvement; Mo, moderately differentiated; Po, poorly differentiated.

## 4 DISCUSSION

This study depicts the incidence of lymph node metastases of Siewert type II AC. To the best of our knowledge, this study possesses the largest sample size with MMN and UMN dissected. The study revealed that metastases in MMN (6.4%) and UMN (6.4%) are relatively low in Siewert type II AC. Even though, the majority of patients with positive MMN or UMN have the number of positive nodes ≤7, suggesting a potential benefit from complete node clearance.

The mapping of LN metastasis for EGJ tumors reported by Kurokawa et al. aroused heated discussion in recent years.^12^, ^16^, ^17^ Significantly, the authors found that metastasis rate of upper mediastinal nodes was up to 10.8% for those with length of esophageal involvement (LEI) ≥ 3 cm.^12^ However, there were only 67 highly selective patients with EGJ AC getting UMN dissected. Besides, unknown number of Siewert type I tumors were encompassed since the research included tumors located 2 cm above to 2 cm below the EGJ. Prospective as the study was, there was an obvious selection bias. MMN and UMN dissection was only performed in patients with LEI >3 cm or with clinically positive node in the upper or middle mediastinum. The researchers predefined LEI >3 cm as a risk factor of UMN metastases based on a retrospective study of very small sample size.^13^ In that retrospective study, only 18 and 33 cases underwent MMN and UMN dissection, respectively. Therefore, the metastases of MMN and UMN in Siewert type II carcinoma still need to be clarified with larger sample size in real‐world clinical practice. In the present study, retrospective as it is, the selection bias is small since MMN and UMN dissection was routinely performed regardless of clinical T stage, clinical N stage, tumor length, or LEI. All 94 cases got comprehensive MMN and UMN dissection.

UMN incorporated station 105, 106tbL, 106recL, and 106recR. The metastasis rate of 105 and 106recL was 0%, which was in line with that of previous reports.^12^, ^18^ The data of Kurokawa et al. showed that the metastasis rate of 106recL and 105 was 1.5% (1/67).^12^ A systematic review reported that the UMN involvement was below 4%.^18^ The metastasis rate of 106tbL was 0% (0/41). As far as we know, this is the first study exploring the metastasis of 106tbL in Siewert type II AC. To sum up, the metastasis rate of 105, 106recL, and 106tbL was extremely low. Therefore, lymph node dissection of these stations is not recommended. In this study, 106recR was only station of UMN that presented with positive nodes. The metastatic rate was 6.4% (6/94), which was comparable to that of 6.0% (4/67) in previous study.^12^ Clinical T stage ≥3, poor differentiation, tumor length >4 cm, and esophageal involvement >3 cm showed higher metastasis rate. The dissection of 106recR was recommended by Kurokawa et al. when LEI >4 cm.^12^ However, in the study 106recR dissection was not performed in patients with LEI ≤3 cm. Thus, the authors did not know the incidence of UMN metastasis in patients with LEI ≤3 cm. Besides, in clinical practice, an accurate measurement of distance from EGJ to proximal margin or epicenter of tumor is often difficult, especially in those of circumferential or large tumors. As a result, in our opinion, it is still debatable to recommend 106recR dissection simply based on LEI. It might be more reasonable to comprehensively take into account clinical T stage, differentiation, tumor length, and LEI when considering 106recR dissection. However, it is worth noting that the overall metastasis rate of 106recR is still low.

As for MMN, the metastasis rate of station 107, 109, and station 108 were 2.1% (2/94) and 5.0% (4/80), respectively, which was in accordance with that of 3.0% (2/67) reported by Kurokawa et al.^12^ Owing to the special anatomical location of station 107, 109, lymphadenectomy in this area is associated with increased surgical trauma and the incidence of postoperative complications.^19^, ^20^ Given the low incidence of positive MMN, routine dissection of which is not recommended.

A randomized, controlled trial comparing the transhiatal and the right transthoracic approaches for Siewert type I and II tumors by a Dutch group revealed that patients with limited positive nodes (1 to 8 positive lymph nodes) could get survival benefit from an extended transthoracic esophagectomy, suggesting that extended lymphadenectomy was beneficial to these patients.^21^, ^22^ In the present study, although the incidence of positive MMN and UMN is low, more than a half of those patients (63.6%, 7/11) had the number of metastatic nodes ≤7. 54.5% (6/11) had pathological N stage ≤2, and 106recR was even found to be the only positive nodes in one patient. These findings are interesting and have never been reported before. The results of the study indicate that positive MMN or UMN does not determine an advanced tumor. On the contrary, most of these patients are of curable disease. This holds paramount importance in clinical practice. Inadequate lymphadenectomy, when performed on patients with a curable disease, represents a regrettable outcome for both the surgeon and the patient. Univariate logistic regression analyses found LEI >3 cm (*p* = 0.042) was related to higher risk for MMN metastases. However, the parameter did not reach a significant level in multivariate analyses (*p* = 0.70). Probably owing to the small sample size (especially that of the positive node group), no independent risk factors were detected mediastinal node metastases. Therefore, it is of vital importance for further studies to distinguish those with higher risk of metastases in MMN and UMN. The enhancement of imaging, which leads to more accurate diagnoses for LNM, is also significant.

This study has several limitations. Firstly, this is a single‐center study with limited sample size, especially for the node‐positive group (11 vs. 83), which introduced obstacles for the study to identify independent risk factors for metastases in MMN or UMN. Secondly, given the short‐term follow‐up, it is unknown whether the dissection of UMN or MMN could bring survival benefit. It is anticipated that continuous follow‐up will yield answers to the question. Moreover, as the patients were from the Department of Thoracic Surgery, some of the Siewert type II EGJ cancer might have not visited our department but visited the abdominal surgical department. Presumably, patients with larger tumor or longer esophageal invasion were treated by esophagectomy, but patients with smaller tumor or shorter esophageal invasion might have been treated by extended gastrectomy by abdominal surgeon. Therefore, the real incidence of the UMMN metastasis might be lower than our data presented.

This study represents the largest study to date exploring metastases of MMN and UMN in Siewert type II AC. Retrospective as it is, the selection bias is small since extended lymphadenectomy is performed routinely. Our findings reveal that the incidence of positive MMN and UMN are relatively low. Thus, routine dissection of these stations is not recommended. LEI >3 cm may be associated with higher risk for mediastinal node metastasis. Certain patients may benefit from extend lymphadenectomy since most of the patients with positive MMN or UMN are with limited number of metastatic nodes.

## AUTHOR CONTRIBUTIONS

**Peng Luo:** Conceptualization (lead); data curation (equal); formal analysis (lead); methodology (equal); project administration (equal); software (equal); supervision (equal); validation (equal); visualization (equal); writing – original draft (lead); writing – review and editing (equal). **Xiankai Chen:** Data curation (lead). **Yafan Yang:** Funding acquisition (lead). **Ruixiang Zhang:** Investigation (lead); methodology (lead). **Xiaozheng Kang:** Funding acquisition (lead). **Jianjun Qin:** Project administration (equal); resources (equal). **Xiuzhu Qi:** Supervision (equal); writing – review and editing (equal). **Yin Li:** Conceptualization (equal); data curation (equal); formal analysis (equal); investigation (equal); methodology (equal); software (equal); supervision (equal); validation (equal); writing – review and editing (equal).

## FUNDING INFORMATION

The authors disclosed receipt of the following financial support for the research, authorship, and/or publication of this article: This study was supported by the Special Program for Basic Resource Survey of the Chinese Ministry of Science and Technology (2019FY101101) and the National Key R&D Program of China (2021YFC2501000).

## CONFLICT OF INTEREST STATEMENT

The authors declare that there is no conflict of interest.

## Data Availability

The datasets used and/or analyzed during the current study are available from the corresponding author upon reasonable request.
